# Health Literacy and Its Sociodemographic Predictors: A Cross-Sectional Study of a Population in Madrid (Spain)

**DOI:** 10.3390/ijerph191811815

**Published:** 2022-09-19

**Authors:** David García-García, Francisco Javier Pérez-Rivas

**Affiliations:** 1 Nursing Primary Health Care Service of Madrid, 28004 Madrid, Spain; 2 Grupo de Investigación UCM “Salud Pública-Estilos de Vida, Metodología Enfermera y Cuidados en el Entorno Comunitario”, Departamento de Enfermería, Facultad de Enfermería, Fisioterapia y Podología, Universidad Complutense de Madrid, 28040 Madrid, Spain; 3 Red de Investigación en Cronicidad, Atención Primaria y Promoción de la Salud—RICAPPS—(RICORS), Instituto de Investigación Sanitaria Hospital 12 de Octubre (imas12), 28041 Madrid, Spain

**Keywords:** health literacy, primary health care, public health, global health, nursing

## Abstract

Background: Health literacy enhances a population’s self-care capacity and helps to reduce health inequalities. This work examines the health literacy of a population attending primary care services and explores its relationship with sociodemographic factors. Methods: This cross-sectional study, conducted at a healthcare center in the Madrid region (Spain), involved adult patients requiring primary care nursing services. One hundred and sixty-six participants were recruited via systematic random sampling. Health literacy was measured using the Health Literacy Questionnaire (HLQ). Results: The studied population showed higher health literacy scores for literacy dimensions 1 (feeling understood and supported by healthcare providers) and 4 (social support for health); the lowest scores were recorded for dimensions 5 (appraisal of health information) and 8 (ability to find good health information). People with a better perceived health status showed a higher level of health literacy. People over 65 years of age, those with an incomplete secondary education, and those who were unemployed returned lower scores for several literacy dimensions. Conclusions: The results contribute to our understanding of the factors that influence health literacy. Identifying the areas in which patients show the poorest health literacy may help us comprehend their needs and better support them.

## 1. Introduction

Poor health literacy (defined in [1,2]) is directly related to higher hospitalization rates, poorer adherence to pharmacological regimens, poorer preventive behavior, a poorer general health status, increased mortality, increased economic costs, and greater health inequalities [3,4]. Low levels of health literacy are therefore a public health problem. Consequently, the World Health Organization included the promotion of health literacy as a key pillar of the 2030 Agenda for Sustainable Development [5]. Indeed, health literacy is now deemed to be an important determinant of health status [3]. Approximately 30% of the population of the European Union is affected by low levels of health literacy—a problem with a likely negative impact on individuals and communities [4].

The first tools for measuring health literacy were the “REALM” [6] (which assesses word recognition) and “TOFHLA” [7] (which measures reading comprehension and numeracy) tests. Later, the “HALS” scale was developed, which incorporates the measurement of dimensions related to health competencies. At present, the most widely used scales are the “European Health Literacy Survey Questionnaire (HLS-EU-Q)”, which consists of 12 dimensions that assess the capacity to access, comprehend, evaluate, and apply information related to health promotion and prevention [8], and the “Health Literacy Questionnaire (HLQ)”, designed to provide healthcare professionals, healthcare institutions, and governments with data that reflect the strengths and weaknesses of individuals and communities in terms of health-related knowledge and skills [1,9].

Health literacy is strongly related to sociodemographic factors such as socioeconomic level and the number of years of education received (the higher the values for these factors, the higher the level of health literacy). Lower levels of health literacy are also commonly related to old age [4]. Associations between health literacy and the extent to which people manage their own health have been found [1]. Some authors indicate that health literacy might help offset the effect of certain social determinants on health, helping to prevent the health inequalities that arise in marginalized populations. However, further testing is needed to confirm this [3,4,10,11].

The aim of the present study was to examine the health literacy of a population attending primary care services in Madrid (Spain), and to explore its relationship with sociodemographic factors.

## 2. Materials and Methods

### 2.1. Study Design and Study Subjects

This cross-sectional study was designed following the STROBE guidelines for observational research [12]. The study population was composed of patients attending the primary care nursing services of the *Centro de Salud Las Águilas*, an urban healthcare center in the Madrid region (Spain). Potential subjects had to be over 18 years of age and willing to participate voluntarily; all those interested gave informed consent to be included. Subjects who could not understand the study or the questionnaire/forms used therein due to language barriers were excluded, as were those suffering an acute process provoking discomfort or a reduction in their capacity to take part. Those who required prompt attention (e.g., those suffering from fever, headache, general discomfort, etc.), and those with cognitive decline or serious mental illness, were also excluded.

The sample size was calculated by conducting a pilot study with 10 patients and calculating their mean (and standard deviation) health literacy. Assuming the population to be infinite, and for an alpha risk of 0.10, a precision of ±0.65 units, a standard deviation of 4.9 units, and no loss to follow-up, a minimum of 154 patients was determined as needed. A total of 166 patients were recruited for the final study. To ensure the validity of the sample, systematic probabilistic sampling was conducted for patients scheduled for care with four nurses: the first selected subject = the first patient on the appointment list, the second subject = fourth on the list, and the third subject = seventh on the list (with three patients maximum recruited per day).

### 2.2. Procedures

The four nurses mentioned above were trained regarding the data collection method. The patients were informed in detail about the study, and all doubts were resolved. The voluntary and altruistic nature of the study was emphasized. The documentation provided to the subjects consisted of the study information sheet, the consent form, the sociodemographic variables data collection sheet, and the HLQ. The information collected was transferred to an Excel file.

The degree of support needed by each patient to complete the questionnaire was assessed. Those who did not require support were transferred to another room where they could complete it without feeling influenced by the presence of the nurse. The self-administration of the questionnaire was an attempt to avoid (as much as possible) any ‘social desirability’ bias. Finished questionnaires were returned and examined to ensure their correct completion. Blank or incoherent responses were reviewed and completed together with the patient to resolve any doubts. When a high degree of support was deemed necessary (e.g., due to impaired vision, illiteracy, etc.), the attending nurse completed the form with the patient following an interview format. When patients had insufficient time to complete the questionnaire at the time of recruitment or needed more help, they were scheduled to return on a different day at a suitable time.

### 2.3. Outcome Measures

The HLQ was used to measure the main variable, i.e., the level of health literacy (measured as a quantitative, discrete variable). The HLQ is a robust questionnaire that was developed following a validity-driven strategy [13]. It has been translated into different languages and adapted to many cultures, and its psychometric properties have been extensively assessed in different samples and populations [9,14,15,16]. Consistent reliability has been reported for all its scales (ranging from 0.77 to 0.90). Testing has shown that its psychometric properties remain consistent with those of the original English version across different contexts [17,18,19,20,21,22,23]. The present study used the Spanish HLQ, which has been tested in primary care settings involving patients on oral anticoagulation treatment to assess the relationship between health literacy and health and treatment outcomes [24], and in patients with cardiovascular diseases to investigate its relationship with social determinants [25]. Unlike other tools, it collects information on health literacy in its entirety, providing a holistic understanding of its definition. It also has a constructivist purpose: the information collected can help determine the actions needed to improve health literacy. The questionnaire analyzes nine dimensions via 44 items, all independent and reliable indicators of health literacy. It consists of two parts: the first evaluates five dimensions related to health literacy via 23 items scored as follows: strongly disagree, disagree, agree, and strongly agree (numerically recorded as a score of 1–4, respectively). The second part evaluates four dimensions via 21 items scored as follows: cannot do or always difficult, usually difficult, sometimes difficult, usually easy, and always easy (numerically recorded as a score of 1–5, respectively). Since the scales collect information on people’s experiences when accessing, using, understanding, and relating to health information and health care services, the results also throw light on the quality of health and social services provided [9].

Information on sociodemographic variable categories such as sex (male or female), age (later defined as ≤65 and >65 years), education (illiterate or incomplete primary education, primary education, secondary education, high school and professional training or university studies), country of birth (Spain or a third country), marital status (single, married, separated or widower), occupation (employee, self-employed, unemployed, retired or pensioner, unpaid domestic work, student or not classifiable), and perceived health status (very bad, bad, fair, good or very good), was also collected.

### 2.4. Statistical Analysis

Following the recommendations for the use of the HLQ, the mean scores for the dimensions—with respect to the different categories of the sociodemographic variables examined—were obtained from the scores of the assessed items. A higher score reflects greater health literacy [11,16]. It should be noted that the HLQ does not provide an overall score for health literacy from the domain scores.

The Kolmogorov-Smirnov test confirmed the health literacy dimension scores to be normally distributed. Thus, differences in the mean scores with respect to the categories of the sociodemographic variables examined were analyzed using the Student’s *t*-test (polytomous sociodemographic variables were transformed into dichotomous variables for this analysis). Significance was set at *p* < 0.05.

The effect size was calculated as Cohen’s d for a standardized difference in means (with 95% confidence intervals). The effect size was considered small for values between >0.20–0.50, medium for 0.50–0.80, and large for >0.80.

Forward stepwise multiple linear regression was also performed to confirm the influence of sociodemographic variables on the scores for the different dimensions.

All calculations were made using the IBM SPSS Statistics 27TM statistical package.

### 2.5. Ethics Approval

The study was approved by the ethics review boards of the Madrid Primary Care Center Assistance Management Research Commission (protocol code 01/22-c approved on 24 January 2022) and by the Ethics Committee of the Complutense University of Madrid (protocol code CE_20220120-10_SAL approved on 20 January 2022).

## 3. Results

### 3.1. Health Literacy Levels

**Figure 1:**
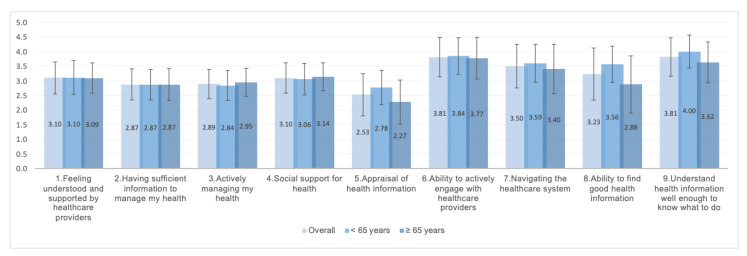
HLQ dimension scores for the study population as a whole and by age group (≤65 and >65 years). Mean and standard deviation for responses to the Health Literacy Questionnaire (HLQ). Dimensions 1–5 were scored on a scale of 1–4, and dimensions 6–9 on a scale of 1–5. Higher score, better health literacy.

For the study population as a whole, the highest health literacy scores were returned for dimensions 1 “feeling understood and supported by healthcare providers” and 4 “social support for health”, and the lowest for dimensions 5 “appraisal of health information” and 8 “ability to find good health information” (Figure 1). Compared to those >65 years of age, the ≤65 age group obtained significantly higher scores for dimensions 5 “appraisal of health information”, 8 “ability to find good health information”, and 9 “understands health information well enough to know what to do”.

### 3.2. Health Literacy and Sociodemographic Characteristics

Dimensions 1 and 2, “feeling understood and supported by healthcare providers” and “having sufficient information to manage my health”, respectively, were not influenced by the sociodemographic characteristics contemplated. However, the following significant differences were found:

- Those with a perceived health status rated as good or very good obtained higher scores for all dimensions than those with a perceived health status of fair, poor, or very poor—except for dimensions 1 “feeling understood and supported by healthcare professionals” and 2 “having sufficient information to manage my health”;
- Those ≤65 years of age had higher scores than those >65 years for dimensions 5 “appraisal of health information”, 8 “ability to find good health information”, and 9 “understands health information well enough to know what to do”;
- Employed subjects had higher scores than those who were unemployed for dimensions 5 “appraisal of health information”, 8 “ability to find good health information”, and 9 “understands health information well enough to know what to do”;
- Those who had completed their secondary education had higher scores than those who had not finished for dimensions 5 “appraisal of health information”, 8 “ability to find good health information”, and 9 “understands health information well enough to know what to do”;
- Those born in Spain had higher scores than those who were born outside the country for dimensions 4 “social support for health” and 6 “ability to actively engage with healthcare providers”;
- For dimension 4 “social support for health”, married subjects returned higher scores than those who were single, separated, or widowed;
- No differences were found in relation to sex.

The largest effect sizes (ES) were seen for dimension 5 “appraisal of health information” with respect to education ES = 0.87 [0.55, 1.21] and for age group ES = −0.74 [−1.06, −0.43]; and for dimension 8 “ability to find good health information” with respect to occupation ES = −0.75 [−1.09, −0.43], education ES = 0.75 [0.44, −1.09], and age group ES = −0.75 [−1.08, −0.45] (Table 1).

### 3.3. Predictive Factors of Health Literacy

The regression model detected no predictors for dimensions 1 “feeling understood and supported by healthcare providers” or 2 “having sufficient information to manage my health”. Sex had no influence on the scores in any dimension.

The variable ‘occupation’ was found to be a positive predictor of the score for dimensions 3 “actively managing my health” and 9 “understand health information enough to know what to do”; those who were employed returned higher scores. An unfinished secondary education was a negative predictive factor for dimensions 5 “appraisal of health information” and 8 “ability to find good health information” and was a positive factor for dimension 4 “social support for health”.

Health status perceived as very bad, bad, or fair was a negative predictor for all dimensions except for dimensions 5 “appraisal of health information” and 8 “ability to find good health information”.

Having been born outside of Spain was a negative predictor for dimensions 4 “social support for health” and 6 “ability to actively engage with healthcare providers”.

Being single, separated, or widowed was a negative predictor for dimensions 4 “social support for health” and 7 “navigating the healthcare system”.

Finally, being aged ≤65 years was a positive predictor for dimensions 5 “appraisal of health information” and 8 “ability to find good health information” (Table 2).

## 4. Discussion

With the exception of dimensions 1” feeling understood and supported by healthcare providers” and 4 “social support for health”, the scores obtained for the examined health literacy dimensions indicate the present population faces several challenges.

In their systematic review, Rajah et al. [26] reported low health literacy to affect between 1.6 and 99.5% of the population, depending on the tool used to measure it. The mean scores reported for almost all HLQ dimensions by Yiu & Bajorek [27] were higher than those recorded in the present study, perhaps because the sample size of the latter study was smaller and reflected a different socioeconomic range. Moreover, the latter study had much more specific inclusion criteria and was performed outside of the European Union. Similarly, Holt et al. [28] reported higher scores for all HLQ dimensions although their results are not as generalizable as those of the present study since their subjects were all young nursing students working towards a degree (and who therefore could be expected to score higher). In contrast, the present scores were higher than those reported by Maindal et al. [18] and Beauchamp et al. [29], respectively, for dimension 1 “feeling understood and supported by healthcare providers” and dimension 4 “social support for health”. The scores reported in an earlier Spanish study [25] are more similar to those of the present work, although those of dimensions 2 “having sufficient information to manage my health”, 3 “actively managing my health”, 5 “appraisal of health information”, 8 “ability to find good health information”, and 9 “understands health information well enough to know what to do” were higher in the present study. The differences between the results of the latter and the present study would appear, therefore, to mainly lie in successful access to, and the use of, health information.

It should be noted that the highest scores in the present study were returned for dimension 1 “feeling understood and supported by health providers” and dimension 4 “social support for health”. It is these that most depend on external factors, specifically on access to health professionals; the high scores recorded might therefore be attributable to the public healthcare system available in Spain and, perhaps, to the patients’ social networks. For the remaining dimensions, the score obtained depends more on each individual’s knowledge, skills, and abilities. Indeed, the lowest score was obtained for dimension 5 “appraisal of health information”. These results suggest that the population is passive in the health environment and that it may not be self-sufficient in seeking and critically analyzing information or in making any related decisions. These results are consistent with those of the above Spanish study that used the HLQ [25] and with those of another study performed in the United Kingdom [30].

When the dimensions were analyzed according to age group, significant differences were observed for dimensions 5 “appraisal of health information”, 8 “ability to find good health information”, and 9 “understands health information well enough to know what to do”, with younger age a positive predictor. The non-appearance of age as a predictive factor with respect to the other dimensions was unexpected. This might indicate the existence of unidentified protective factors (perhaps the perceived health status) that allow for good scores. Dimensions 5 “appraisal of health information”, 8 “ability to find good health information”, and 9 “understands health information well enough to know what to do” are those for which a certain level of literacy is needed if a high score is to be obtained [10]. The latter is the most complex dimension of all, and the younger age group returned the highest scores. This confirms the influence of age on health literacy and agrees with the results of other studies [4,25,31,32,33,34,35]. It may be that old age is associated with greater passivity and/or difficulty in interacting with health information and the health environment, with certain responsibilities for care being left to the social support network, including family, caregivers, and health professionals. In contrast, Svendsen et al. [35] reported the lowest health literacy to exist among younger people and the highest among older people, although this might have been due to the age groups in their study being more unbalanced (with many more younger subjects than older ones). Maindal et al. [18], who specifically used the HLQ in their study, found no significant age-related differences except with respect to dimension 4 “social support for health” (with younger subjects scoring higher). This may have been due to their recruiting a slightly younger sample. In addition, Beauchamp et al. [29] identified age-related HQL score differences only for dimensions 3 “actively managing my health” and 4 “social support for health”. However, a study conducted in Spain [25] reported the same differences as those reported here for dimensions 5 “appraisal of health information”, 8 “ability to find good health information”, and 9 “understands health information well enough to know what to do”, as well as for dimension 7 “navigating the healthcare system”.

In agreement with that reported by other authors [32,33], sex had no influence on any dimension score. Males are reported to be more health literate than females in some studies [32], while males are significantly less health literate in others [34,35]. Cabellos-García et al. [25] reported higher scores for males with respect to dimensions 8 “ability to find good health information” and 9 “understands health information well enough to know what to do”, as did Beauchamp et al. [29] with respect to dimensions 4 “social support for health” and 6 “ability to actively engage with healthcare providers”. Maindal et al. [18], in contrast, reported higher scores for females with respect to dimensions 8 “ability to find good health information” and 9 “understands health information well enough to know what to do”.

In agreement with the current literature, educational level and health literacy appeared positively related [5,10,25,26,30,34,35,36]. In all these studies, those who had completed secondary education returned significantly higher scores for dimensions 5 “appraisal of health information”, 8 “ability to find good health information”, and 9 “understands health information well enough to know what to do” compared to those who had not finished it. Indeed, the present regression analysis revealed an incomplete secondary education to be a negative predictor for dimensions 5 and 8, and a positive predictor for dimension 4. An unfinished secondary education appeared as a positive predictor for dimension 4 “social support for health”, implying that, in the present study, the health service provided good support, especially for more vulnerable people. When studies that specifically used the HLQ were examined, the exact same differences [29], or very similar ones [18], were found. Cabellos-García et al. [25] found educational level to have a significant impact on the scores for all dimensions.

Subjects in the employed group scored higher than those in the unemployed group for dimensions 5 “appraisal of health information”, 8 “ability to find good health information”, and 9 “understands health information well enough to know what to do”. No other studies have examined differences according to occupation.

It should be noted that the same differences were seen for age group, educational level, and occupation with respect to dimensions 5 “appraisal of health information”, 8 “ability to find good health information”, and 9 “understand health information well enough to know what to do”. This supports the idea that these more complex dimensions depend strongly on socioeconomic status.

Being born in Spain or elsewhere generated differences with respect to dimensions 4 “social support for health” and 6 “ability to actively engage with healthcare providers”, with the latter group returning poorer scores. Indeed, the regression analysis determined being born outside of Spain to be a negative predictor for these dimensions. While Guggiari et al. [33] found no significant differences in this respect, Svendsen et al. [35] reported findings similar to those of the present work. Baccolini et al. [4] suggest that cultural differences can translate into different beliefs and attitudes that influence health literacy. In a study that specifically used the HLQ [29], results similar to those of the present work were reported. It may be that the foreign-born population may experience vulnerabilities that impair access to health information and services, thus helping to maintain or even worsen health inequalities. It should be remembered too, that migrants often arrive in new countries without their relatives, and may therefore have less family and social support with respect to their health (dimension 4 “social support for health”). They may also interact less with the health system (sometimes simply because they do not know how it works), limiting their chances of scoring highly for dimension 6 “ability to actively engage with healthcare providers”. It is important to note, however, that the sample examined in the present study had a very small number of foreign-born subjects and that these, in any event, came from countries where Spanish is spoken; this limits the generalization of any interpretations.

A clear, positive relationship was observed between perceived health status and health literacy. Those who perceived their health as good or very good scored better for all dimensions except for dimensions 1 “feeling understood and supported by healthcare providers” and 2 “having sufficient information to manage my own health”. The regression model showed health perceived as very bad, bad, or fair to be a negative predictor for all dimensions except for 5 “appraisal of health information” and 8 “ability to find good health information”. Other studies have reported a strong correlation to exist between the assessment of one’s own health and level of health literacy [1,32]. Maindal et al. [18], who used the HLQ questionnaire, identified the same influence of perceived health status on all dimensions.

Single, separated, and widowed people returned significantly lower scores for dimension 4 “social support for health”; these factors were negative predictors for this dimension as well as for dimension 7 “navigating the healthcare system”. The fact that these people largely live alone may mean they have less family and social support in matters related to health. Other authors have reported similar findings [34]. However, Azlan et al. [32] found no such differences with respect to marital status, although this might be explained by the relatively young age of their sample. The literature contains no similar reports that specifically used the HLQ to examine the effect of this variable.

It should be noted that, in relation to the sociodemographic variables examined, no differences were found in dimensions 1, “feeling understood and supported by healthcare providers” or 2, “having sufficient information to manage my health”.

Maindal et al. [18] reported perceived health status to influence scores for dimensions 1 “feeling understood and supported by healthcare providers” and 2 “having sufficient information to manage my health”. The latter was also influenced by education received and long illness or disability. Beauchamp et al. [29] reported birth country and whether English was spoken at home as influencing scores for dimension 1, and the number of chronic diseases and whether English was spoken at home as influencing those for dimension 2, “having sufficient information to manage my health”. Variables such as the number of chronic diseases, disabilities, and polypharmacy should therefore be included in future research on health literacy.

The present study suffers from the limitation of being cross-sectional; while associations can be detected, causality cannot be proved. In addition, since the study was performed at a single center that attended to a population of medium-low socioeconomic level, the results might only be generalizable to health areas of the same characteristics. Certainly, the results for the foreign-born population should be understood with caution due to the small subsample available. Additionally, the data were collected after the critical period of the COVID-19 pandemic, which may have had repercussions on the study sample’s knowledge and health beliefs. Nonetheless, this work made use of a rigorous sampling technique and determined the required sample size after considering biases, which greatly strengthens the internal validity of the results. In addition, the inclusion and exclusion criteria were not very restrictive, allowing for a heterogeneous sample of subjects, thus increasing the external validity of the results.

Future research should focus on analyzing the relationship between social context and behavior as a determinant of health literacy and on health education programs designed to improve health literacy.

## 5. Conclusions

The present study contributes to our understanding of the factors that influence health literacy and identifies areas in which patients are most vulnerable. This information could be used by healthcare providers and other stakeholders to promote adequate levels of health literacy and thus help prevent health inequalities, encourage the responsible use of healthcare resources, and empower the users of the healthcare system.

## Figures and Tables

**Table 1:** Relationship between sociodemographic variables and HLQ scores.

Variable	1. Feeling Understood and Supported by Healthcare Providers	2. Having Sufficient Information to Manage My Own Health	3. Actively Managing My Health	4. Social Support for Health	5. Appraisal of Health Information	6. Ability to Actively Engage with Healthcare Providers	7. Navigating the Healthcare System	8. Ability to Find Good Health Information	9. Understands Health Information Enough to Know What to Do
**Age group**									
<65 years									
Mean (SD)	3.11 (0.59) n = 81	2.88 (0.53) n = 81	2.83 (0.52) n = 81	3.07 (0.54) n = 81	**2.79 (0.60)** n = 81	3.84 (0.62) n = 81	3.60 (0.65) n = 81	**3.55 (0.64)** n = 81	**4.00 (0.57)** n = 81
≥65 years									
Mean (SD)	3.09 (0.52) n = 85	2.86 (0.54) n = 85	2.95 (0.47) n = 85	3.12 (0.49) n = 85	**2.29 (0.74)** n = 85	3.77 (0.72) n = 85	3.41 (0.83) n = 85	**2.92 (0.98)** n = 85	**3.64 (0.69)** n = 85
*t*-test	F = 0.645; *p* = 0.844	F = 0.006; *p* = 0.859	F = 3.034; *p* = 0.133	F = 1.310; *p* = 0.478	F = 9.109; ***p* = 0.000**	F = 1.439; *p* = 0.470	F = 4.425; 164; *p* = 0.117	F = 17.304; ***p* = 0.000**	F = 4.287; ***p* = 0.000**
Effect Size (95% CI)	−0.04 (−0.34, 0.27)	−0.04 (−0.34, 0.27)	0.24 (−0.06, 0.55)	0.10 (−0.21, 0.40)	−0.74 (−1.06, −0.43)	−0.10 (−0.41, 0.20)	−0.25 (−0.56, 0.05)	−0.75 (−1.08, −0.45)	−0.56 (−0.89, −0.26)
**Sex**									
Male									
Mean (SD)	3.13 (0.50) n = 75	2.91 (0.54) n = 75	2.85 (0.51) n = 75	3.14 (0.49) n = 75	2.59 (0.73) n = 75	3.88 (0.60) n = 75	3.57 (0.70) n = 75	3.20 (0.85) n = 75	3.85 (0.64) n = 75
Female									
Mean (SD)	3.07 (0.59) n = 91	2.84 (0.53) n = 91	2.92 (0.49) n = 91	3.06 (0.53) n = 91	2.48 (0.71) n = 91	3.75 (0.72) n = 91	3.44 (0.79) n = 91	3.25 (0.92) n = 91	3.79 (0.68) n = 91
*t*-test	F = 0.456; *p* = 0.532	F = 0.011; *p* = 0.409	F = 0.734; *p* = 0.371	F = 0.196; *p* = 0.338	F = 0.107; *p* = 0.318	F = 3.475; *p* = 0.225	F = 1.024; *p* = 0.273	F = 0.916; *p* = 0.718	F = 0.253; *p* = 0.580
Effect Size (95% CI)	−0.11 (−0.42, 0.20)	−0.13 (−0.44, 0.18)	0.14 (−0.17, 0.45)	−0.16 (−0.47, 0.15)	−0.15 (−0.46, 0.15)	−0.19 (−0.50, 0.11)	−0.17 (−0.48, 0.13)	0.06 (−0.25, 0.36)	−0.09 (−0.40, 0.22)
**Occupation**									
Employed									
Mean (SD)	3.08 (0.63) n = 61	2.89 (0.50) n = 61	2.79 (0.53) n = 61	3.10 (0.52) n = 61	**2.74 (0.60)** n = 61	3.87 (0.62) n = 61	3.59 (0.67) n = 61	**3.62 (0.57)** n = 61	**4.05 (0.54)** n = 61
Unemployed									
Mean (SD)	3.10 (0.50) n = 103	2.85 (0.56) n = 103	2.95 (0.47) n = 103	3.09 (0.51) n = 103	**2.40 (0.76)** n = 103	3.75 (0.70) n = 103	3.44 (0.79) n = 103	**2.99 (0.96)** n = 103	**3.67 (0.69)** n = 103
*t*-test	F = 2.207; *p* = 0.798	F = 0.296; *p* = 0.722	F = 2.286; *p* = 0.052	F = 0.292; *p* = 0.912	F = 7.845; ***p* = 0.003**	F = 0.816; *p* = 0.294	F = 2.531; *p* = 0.208	F = 17.189; ***p* = 0.000**	F = 3.249; ***p* = 0.000**
Effect Size (95% CI)	0.04 (−0.28, 0.36)	−0.07 (−0.39, 0.24)	0.32 (0.01, 0.65)	−0.02 (−0.34, 0.30)	−0.48 (−0.81, −0.16)	−0.18 (−0.50, 0.14)	−0.20 (−0.52, 0.12)	−0.75 (−1.09, −0.43)	−0.59 (−0.93, −0.27)
**Studies**									
Unfinished secondary education									
Mean (SD)	3.12 (0.53) n = 66	2.88 (0.56) n = 66	2.91 (0.52) n = 66	3.19 (0.48) n = 66	**2.18 (0.79)** n = 66	3.78 (0.75) n = 66	3.43 (0.86) n = 66	**2.85 (1.05)** n = 66	**3.61 (0.75)** n = 66
Completed secondary education									
Mean (SD)	3.09 (0.56) n = 100	2.86 (0.51) n = 100	2.88 (0.48) n = 100	3.04 (0.50) n = 100	**2.76 (0.57)** n = 100	3.83 (0.62) n = 100	3.55 (0.67) n = 100	**3.48 (0.65)** n = 100	**3.95 (0.55)** n = 100
*t*-test	F = 0.015; *p* = 0.718	F = 0.157; *p* = 0.790	F = 0.041; *p* = 0.723	F = 0.345; *p* = 0.057	F = 16.794; ***p* = 0.000**	F = 3.608; *p* = 0.643	F = 4.418; *p* = 0.346	F = 28.312; ***p* = 0.000**	F = 9.330; ***p* = 0.001**
Effect Size (95% CI)	−0.05 (−0.37, 0.26)	−0.04 (−0.35, 0.28)	−0.06 (−0.37, 0.25)	−0.30 (−0.62, 0.01)	0.87 (0.55, 1.21)	0.07 (−0.24, 0.39)	0.16 (−0.15, 0.47)	0.75 (0.44, 1.09)	0.53 (0.22, 0.86)
**Perceived health status**									
Very bad, bad, fair									
Mean (SD)	3.04 (0.45) n = 65	2.82 (0.47) n = 65	**2.74 (0.49)** n = 65	**2.99 (0.49)** n = 65	**2.33 (0.70)** n = 65	**3.62 (0.63)** n = 65	**3.32 (0.67)** n = 65	**2.98 (0.91)** n = 65	**3.62 (0.64)** n = 65
Good, very good									
Mean (SD)	3.14 (0.60) n = 101	2.90 (0.57) n = 101	**2.99 (0.48)** n = 101	**3.17 (0.51)** n = 101	**2.66 (0.70)** n = 101	**3.93 (0.68)** n = 101	**3.62 (0.78)** n = 101	**3.39 (0.84)** n = 101	**3.94 (0.64)** n = 101
*t*-test	F = 6.662; *p* = 0.202	F = 2.575; *p* = 0.361	F = 1.263; ***p* = 0.001**	F = 0.401; ***p* = 0.024**	F = 0.181; ***p* = 0.004**	F = 0.488; ***p* = 0.004**	F = 1.195; ***p* = 0.012**	F = 0.553; ***p* = 0.003**	F = 0.050; ***p* = 0.002**
Effect Size (95% CI)	0.18 (−0.13, 0.50)	0.15 (−0.16, 0.47)	0.51 (0.20, 0.84)	0.36 (0.05, 0.68)	0.47 (0.16, 0.79)	0.47 (0.16, 0.79)	0.40 (0.09, 0.73)	0.47 (0.16, 0.79)	0.50 (0.19, 0.82)
**Birth country**									
Spain									
Mean (SD)	3.12 (0.54) n = 151	2.89 (0.52) n = 151	2.89 (0.50) n = 151	**3.13 (0.49)** n = 151	2.51 (0.74) n = 151	**3.84 (0.67)** n = 151	3.51 (0.76) n = 151	3.20 (0.90) n = 151	3.81 (0.67) n = 151
Foreign country									
Mean (SD)	2.90 (0.63) n = 15	2.67 (0.61) n = 15	2.93 (0.53) n = 15	**2.74 (0.56)** n = 15	2.76 (0.48) n = 15	**3.43 (0.55)** n = 15	3.39 (0.64) n = 15	3.52 (0.61) n = 15	3.89 (0.48) n = 15
*t*-test	F = 0.022; *p* = 0.143	F = 0.591; *p* = 0.123	F = 0.009; *p* = 0.738	F = 0.274; ***p* = 0.005**	F = 5.538; *p* = 0.081	F = 0.015; ***p* = 0.022**	F = 0.495; *p* = 0.555	F = 3.207; *p* = 0.180	F = 2.135; *p* = 0.643
Effect Size (95% CI)	−0.40 (−0.94, 0.13)	−0.41 (−0.96, 0.12)	0.08 (−0.45, 0.61)	−0.78 (−1.33, −0.25)	0.34 (−0.19, 0.89)	−0.62 (−1.16, −0.09)	−0.16 (−0.70, 0.37)	0.36 (−0.17, 0.90)	0.12 (−0.41, 0.66)
**Marital status**									
Single, separated, widower									
Mean (SD)	3.05 (0.58) n = 78	2.83 (0.54) n = 78	2.84 (0.57) n = 78	**3.01 (0.55)** n = 78	2.48 (0.72) n = 78	3.75 (0.64) n = 78	3.38 (0.83) n = 78	3.20 (0.89) n = 78	3.74 (0.69) n = 78
Married									
Mean (SD)	3.14 (0.52) n = 88	2.90 (0.53) n = 88	2.94 (0.42) n = 88	**3.17 (0.46)** n = 88	2.57 (0.72) n = 88	3.86 (0.70) n = 88	3.61 (0.66) n = 88	3.25 (0.89) n = 88	3.88 (0.63) n = 88
*t*-test	F = 0.136; *p* = 0.267	F = 0.000; *p* = 0.365	F = 8.877; *p* = 0.184	F = 2.104; ***p* = 0.044**	F = 0.100; *p* = 0.397	F = 1.147; *p* = 0.292	F = 2.681; *p* = 0.051	F = 0.037; *p* = 0.748	F = 0.685; *p* = 0.190
Effect Size (95% CI)	0.16 (−0.14, 0.47)	0.13 (−0.18, 0.44)	0.20 (−0.10, 0.51)	0.14 (−0.17, 0.45)	0.12 (−0.18, 0.43)	0.16 (−0.14, 0.47)	0.31 (0.00, 0.62)	0.06 (−0.25, 0.36)	0.21 (−0.09, 0.52)

Health literacy questionnaire (HLQ), standard deviation (SD), confidence interval (CI). Results in bold are significantly different to one another (*p* < 0.05); Student *t*-test); Effect size calculated using Cohen’s d for standardised difference in means. Interpretation of ES: “small” >0.20−0.50 SD, “medium” 0.50−0.80 SD, and “large” >0.80 SD.

**Table 2:** Results of multiple regression analysis: positive and negative predictors of health literacy.

Predictors	Beta	*p* Value
**Dimension 3** **Actively managing my health** R 0.345/R^2^ 0.119/adjusted R^2^ 0.108/F10.856		
Constant	2.865 (2.741, 2.989)	0.000
Occupation: unemployed	0.240 (0.085, 0.395)	0.003
Health perceived status: very bad/bad/fair	−0.326 (−0.480, −0.172)	0.000
**Dimension 4** **Social support for health** R 0.373/R^2^ 0.139/adjusted R^2^ 117/F6.424		
Constant	3.225 (3.091, 3.358)	0.000
Secondary education: unfinished	0.176 (0.018, 0.334)	0.030
Birth country: foreign country	−0.332 (−0.594, −0.070)	0.013
Health perceived status: very bad/bad/fair	−0.246 (−0.402, −0.091)	0.002
Marital status: single/separated/widower	−0.170 (−0.317, −0.022)	0.025
**Dimension 5** **Appraisal of health information** R 0.418/R^2^ 0.175/adjusted R^2^ 0.165/F17.078		
Constant	2.573 (2.352, 2.793)	0.000
Age group: <65 years	0.257 (0.010, 0.505)	0.041
Secondary education: unfinished	−0.426 (−0.678, −0.174)	0.001
**Dimension 6** **Ability to actively engage with healthcare providers** R 0.291/R^2^ 0.085/adjusted R^2^ 0.073/F7.452		
Constant	3.961 (3.828, 4.093)	0.000
Birth country: foreign country	−0.428 (−0.774, −0.082)	0.016
Health perceived status: very bad/bad/fair	−0.319 (−0.523, −0.114)	0.002
**Dimension 7** **Navigating the healthcare system** R 0.255/R^2^ 0.065/adjusted R^2^ 0.053/F5.602		
Constant	3.732 (3.551, 3.913)	0.000
Health perceived status: very bad/bad/fair	−0.312 (−0.544, −0.080)	0.009
Marital status: single/separated/widower	−0.241 (−0.468, −0.015)	0.037
**Dimension 8** **Ability to find good health information** R 0.396/R^2^ 0.157/adjusted R^2^ 0.146/F14.946		
Constant	3.181 (2.907, 4.455)	0.000
Age group: <65 years	0.413 (0.105, 0.720)	0.009
Secondary education: unfinished	−0.386 (−0.700, −0.073)	0.016
**Dimension 9** **Understands health information enough to know what to do** R 0.333/R^2^ 0.111/adjusted R^2^ 0.100/F10.032		
Constant	3.787 (3.630, 3.944)	0.000
Occupation: employed	0.323 (0.116, 0.529)	0.002
Health perceived status: very bad/bad/fair	−0.245 (−0.450, −0.040)	0.019

## Data Availability

The data presented in this study area is available upon request from the corresponding author.
